# Translation and Cultural Adaptation of the Home Graded Repetitive Arm Supplementary Program (Home-GRASP) for Stroke Survivors in Spain

**DOI:** 10.3390/healthcare14203418

**Published:** 2026-10-13

**Authors:** Carlos Soler-Pons, Empar Casaña-Escriche, Pablo García-Millán, Miriam Hurtado-Pomares, Desirée Valera-Gran, Eva María Navarrete-Muñoz

**Affiliations:** 1 La Pedrera Hospital, 03700 Denia, Spain; carlossolerpons@gmail.com; 2 Occupational Therapy Research Group (InTeO, Investigación en Terapia Ocupacional), Miguel Hernández University, 03550 San Juan de Alicante, Spain; ecasana@umh.es (E.C.-E.); mhurtado@umh.es (M.H.-P.); enavarrete@umh.es (E.M.N.-M.); 3 Institute for Health and Biomedical Research of Alicante (ISABIAL, Instituto de Investigación Sanitaria y Biomédica de Alicante), 03010 Alicante, Spain; 4 Fundación Hospitalarias Valencia, 46011 Valencia, Spain; pablo.garcia@fundacionhospitalarias.org

**Keywords:** stroke, home-based rehabilitation, upper extremity, occupational therapy, cross-cultural adaptation, translation, ease of use, Home-GRASP

## Abstract

**Highlights:**

**What are the main findings?**

- The Home-GRASP participant exercise manual was successfully translated and cross-culturally adapted into Spanish while preserving the content and therapeutic intent of the original program.
- Pilot testing with stroke survivors in Spain provided preliminary evidence supporting the clarity, comprehensibility, and ease of use of the adapted manual, with only minor revisions required to optimize exercise instructions and visual materials.

**What are the implications of the main findings?**

- The Spanish version of the Home-GRASP manual provides a culturally adapted resource that may support home-based upper-limb rehabilitation for stroke survivors in Spain.
- Clear and accessible instructional materials, combined with initial professional guidance, may facilitate safe implementation and engagement in home-based rehabilitation programs after stroke.

**Abstract:**

**Background/Objectives**: Home-based rehabilitation programs can increase upper-limb practice after stroke and support recovery beyond formal therapy sessions. However, no Spanish version of the Home Graded Repetitive Arm Supplementary Program (Home-GRASP) participant exercise manual was available. This study aimed to translate and cross-culturally adapt the manual for use in Spain and evaluate its clarity, comprehensibility, and ease of use. **Methods**: The original Home-GRASP participant exercise manual was independently translated into Spanish by two translators, reviewed by an expert committee, culturally adapted, and pilot tested with 13 adults with subacute or chronic stroke-related upper-limb hemiparesis receiving outpatient rehabilitation in Spain. Clarity and comprehensibility were assessed through direct observation of exercise performance and a participant questionnaire. **Results**: The expert committee considered that forward translations preserved the underlying concepts and therapeutic intent of the original manual and required only minor linguistic modifications. Expert review resulted in adaptations to photographs, exercise instructions, and layout. During pilot testing, all 35 exercises were evaluated, resulting in 49 exercise performances. Seven execution errors were identified across four exercises and were mainly related to ambiguities in movement descriptions or visual instructions. Following revision of the affected exercises, no further errors were observed. All participants reported that the manual was easy to use, 92.3% found it easy to understand, and 76.9% reported being able to perform the exercises independently. **Conclusions**: The Spanish version of the Home-GRASP participant exercise manual was successfully translated and cross-culturally adapted for use in Spain. The adapted manual showed preliminary evidence of satisfactory clarity, comprehensibility, and ease of use, supporting its use as a culturally appropriate resource for home-based upper-limb rehabilitation after stroke.

## 1. Introduction

Stroke is a sudden injury to the brain that can result in a wide range of sensory, motor, cognitive, emotional, and behavioral impairments that often limit an individual’s ability to perform activities of daily living (ADLs) [1]. Upper limb impairment is among the most common and disabling consequences of stroke, affecting approximately 48–60% of stroke survivors [2]. However, only 15–20% of affected individuals achieve full functional recovery of the upper extremity [3]. Restoring upper limb function is essential for maintaining independence in daily activities and community participation. Previous studies have shown that persistent upper extremity motor impairment is strongly associated with reduced societal participation and poorer health-related quality of life after stroke [4,5]. Consequently, improving upper limb function remains a major rehabilitation goal and ongoing challenge in stroke care [3].

Increasing the dose of upper limb rehabilitation has been consistently associated with improved motor recovery and functional outcomes after stroke [6,7]. However, delivering high-intensity rehabilitation often requires additional therapeutic resources, specialized equipment, or extended treatment time, which may be difficult to implement in routine clinical practice. Limited availability of rehabilitation professionals, logistical constraints within healthcare services, and financial barriers may restrict access to intensive therapy for many stroke survivors [7].

Evidence from stroke rehabilitation settings suggests that the amount of rehabilitation received is frequently lower than recommended. Bernhardt et al. reported that individuals in inpatient stroke units were engaged in therapeutic activities for only 5.3% of the day, with upper limb practice accounting for just 4–11 min daily, whereas more than 60% of their time was spent resting alone [8]. These findings highlight a substantial amount of underutilized time that could potentially be devoted to additional therapeutic practice beyond scheduled rehabilitation sessions. Furthermore, interventions based on intensive, repetitive, and task-oriented practice have shown the strongest evidence for promoting upper-limb recovery following stroke [9]. Home-based rehabilitation programs may facilitate the delivery of this additional practice, promote the transfer of therapeutic gains to everyday activities, and address limitations in access to rehabilitation services after hospital discharge [3,9]. Consequently, home-based rehabilitation programs have emerged as a promising strategy to increase therapy dosage and optimize recovery following stroke [3,10].

The Graded Repetitive Arm Supplementary Program (GRASP) is a self-administered upper-limb exercise program designed for stroke survivors and supervised by an occupational therapist [11]. It comprises a manual containing 35 exercises targeting different components of upper-limb rehabilitation and is based on the principles of high-intensity practice, strengthening, repetition, and task-oriented training [11]. Participants are encouraged to complete an additional hour of practice each day over an eight-week period, thereby increasing rehabilitation dosage without substantially increasing the cost of therapy [11]. The program requires only low-cost equipment, such as therapy balls, hand grippers, and wrist weights, together with commonly available household items. Evidence supports the effectiveness of GRASP in improving upper-limb function following stroke [11]. In addition, qualitative research has shown that stroke survivors perceive GRASP as a valuable and engaging rehabilitation program, with potential for implementation during the early stages of stroke recovery [12].

To extend the delivery of intensive, repetitive, and task-oriented upper-limb practice beyond clinical settings, the Home Graded Repetitive Arm Supplementary Program (Home-GRASP) program was developed to facilitate the continuation of rehabilitation at home through a structured exercise program [13]. Initial findings showed the feasibility and acceptability of Home-GRASP among community-dwelling stroke survivors following completion of formal rehabilitation, [13] while more recent research has also supported the feasibility of the Home-GRASP in individuals living with chronic stroke [14].

At the time of this study, no Spanish version of the Home-GRASP participant exercise manual was available. The cross-cultural adaptation of rehabilitation interventions is essential to ensure that instructions, terminology, and examples are linguistically accurate, culturally relevant, and understandable for the target population [15]. Cross-cultural adaptation may require modifications to wording, content, format, or delivery procedures to maintain the intent and ease of use of the original intervention while ensuring its relevance within a new cultural context [15,16]. Therefore, the aim of this study was to translate and culturally adapt the Home-GRASP manual for use in Spain and to evaluate the clarity and practical comprehensibility of the adapted instructions among stroke survivors.

## 2. Materials and Methods

### 2.1. Study Design

This study was conducted as part of the development phase of REC-HOMEGRASP randomized clinical trial (ClinicalTrials.gov Identifier: NCT07146789). It involved the translation and cross-cultural adaptation of the Home-GRASP participant exercise manual, followed by pilot testing with stroke survivors in Spain presenting upper-limb hemiparesis to assess clarity and comprehensibility, and ease of use of the Spanish version.

### 2.2. Participants

Participants were recruited from the Brain Injury Unit of Fundación Hospitalarias (Valencia, Spain). Individuals were eligible if they were aged 18 years or older, had experienced a stroke at least three months previously with resulting upper-limb hemiparesis, were receiving outpatient rehabilitation, were proficient in Spanish, and presented active movement in all joints of the affected upper limb, as determined from medical records and clinical assessment by the treating therapist. No standardized motor assessment threshold was applied as part of the eligibility criteria. Participants were excluded if they had cognitive impairment (Mini-Mental State Examination score < 21), excessive pain in the affected upper limb (Visual Analogue Scale > 7), severe visual or hearing impairments that prevented participation in the study procedures, or upper-limb amputation.

A total of 13 stroke survivors participated in the pilot-testing phase. Sociodemographic and clinical data were collected, including sex, age, educational level, affected upper limb, and time since stroke.

### 2.3. Home-GRASP Program

The Home Graded Repetitive Arm Supplementary Program (Home-GRASP) was developed by Janice Eng and colleagues at the University of British Columbia, Canada (https://neurorehab.med.ubc.ca/grasp/; accessed on 2 June 2026). Home-GRASP is an 8-week home-based intervention designed to increase upper-limb training among stroke survivors with upper-limb hemiparesis. The program was derived from the original GRASP intervention [11] and incorporates graded exercises that promote upper-limb strength, mobility, coordination, dexterity, and functional task performance through repetitive, task-oriented training [13].

The program is supported by a participant exercise manual organized into four main sections: (1) an introduction to the program and its objectives; (2) required materials and general recommendations for participation; (3) a set of 35 progressive exercises targeting different aspects of upper-limb function; and (4) daily and weekly logs used to document participation and monitor adherence. The exercises are completed using inexpensive materials and common household objects and can be adapted to match each participant’s functional abilities, allowing the level of challenge to be progressively increased over time [13].

Participants are expected to complete approximately one hour of home-based training per day, seven days per week, over an eight-week period [13]. Before beginning the program, an occupational therapist provides individualized instruction on the use of the manual, exercise performance, and the materials required for participation. Participants document their sessions using the logs included in the manual. Weekly follow-up is provided to support adherence, address potential difficulties, and adjust the exercise program according to individual progress [13].

### 2.4. Translation and Cross-Cultural Adaptation Procedure

Permission to translate and culturally adapt the Home-GRASP participant exercise manual into Spanish was obtained from the program developer prior to the start of the study. The adaptation process was conducted following internationally recognized recommendations for the cross-cultural adaptation of health-related instruments and interventions [17,18]. Consistent with contemporary methodological recommendations, back-translation was not included in the adaptation process. Although back-translation is incorporated in some traditional frameworks, evidence suggests that expert committee review and testing with representatives of the target population may contribute more substantially to ensuring conceptual equivalence and identifying adaptation issues than back-translation alone [19,20].

**Figure 1:**
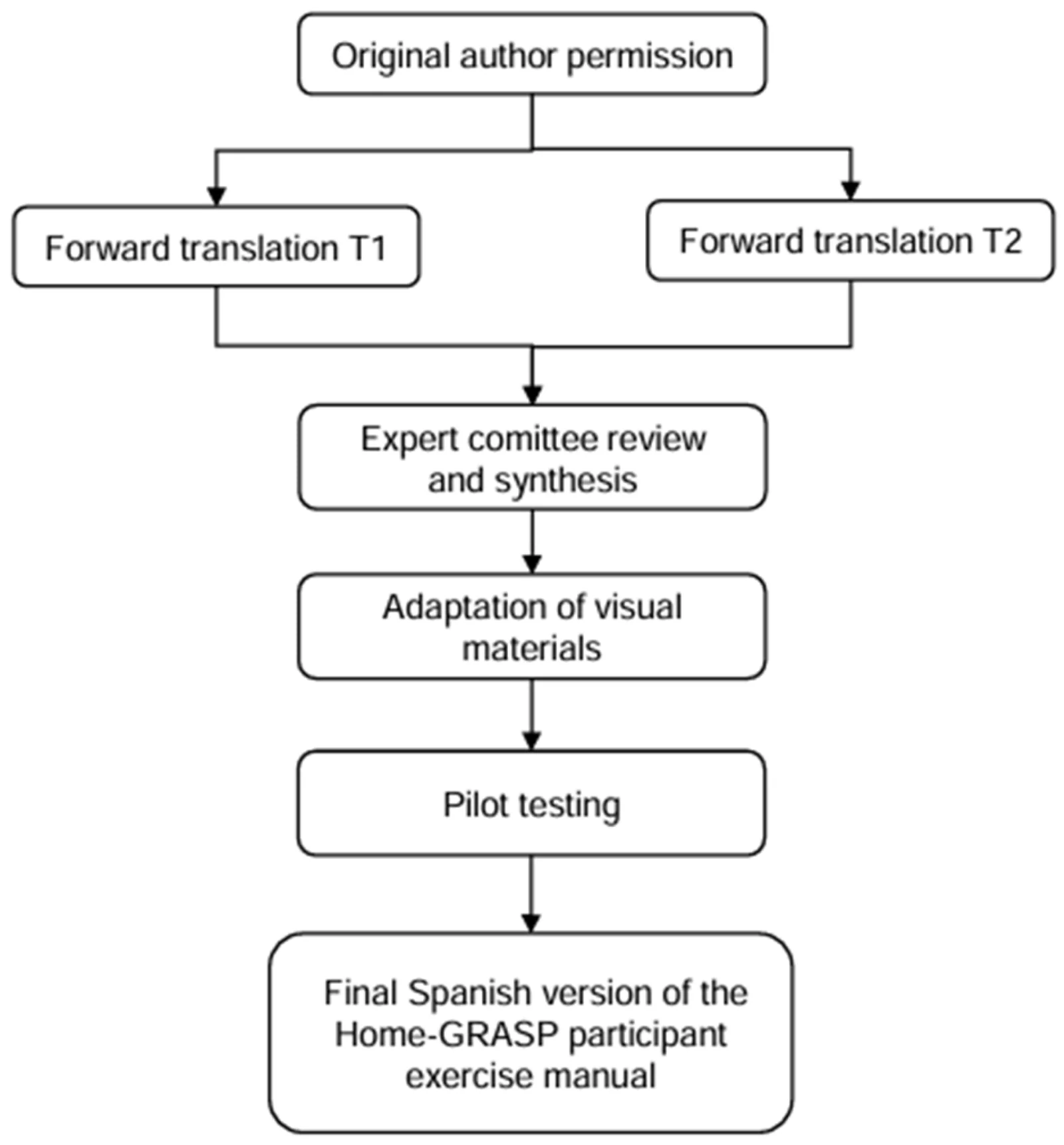
Stages of the Translation and Cross-Cultural Adaptation of the Home-GRASP Participant Exercise Manual into Spanish.

The process comprised five sequential phases: forward translation, expert committee review, adaptation of visual materials, pilot testing, and development of the final Spanish version. Figure 1 provides an overview of the translation and cross-cultural adaptation process undertaken to develop the Spanish version of the Home-GRASP participant exercise manual.

#### 2.4.1. Step 1. Forward Translation

Two independent translators (T1 and T2), both native Spanish speakers fluent in English, independently translated the original Home-GRASP participant exercise manual from English into Spanish. T1 was a professional bilingual translator without a clinical background, whereas T2 was an occupational therapist with experience in stroke rehabilitation and familiarity with the Home-GRASP program. This combination was intended to provide both linguistic expertise and clinical insight into the content and objectives of the intervention. The aim of this phase was to produce conceptually equivalent translations [17,18].

#### 2.4.2. Step 2. Expert Committee Review

An expert committee reviewed and synthesized the two forward translations into a single preliminary Spanish version. The committee comprised five occupational therapists with more than five years of clinical experience in stroke rehabilitation and two researchers with expertise in the translation and cross-cultural adaptation of health-related instruments. All committee members were native Spanish speakers. Committee members were selected on the basis of their clinical and methodological expertise relevant to the adaptation of the Home-GRASP manual. Discrepancies between translations were discussed and resolved through consensus, with particular attention paid to semantic, idiomatic, conceptual, and experiential equivalence [17,18]. Expert committee meetings were video recorded and subsequently reviewed by members of the research team to document and summarize the translation issues identified and the decisions reached during the synthesis process.

The committee also reviewed the clarity, readability, and cultural relevance of the translated content to ensure its suitability for stroke survivors in the Spanish context.

#### 2.4.3. Step 3. Adaptation of Visual Materials

The visual content of the original manual was also reviewed and adapted as part of the overall adaptation process. The photographs included in the English version were replaced to ensure consistency with the linguistic, contextual, and visual adaptations introduced in the Spanish version. The expert committee considered that the photographs should be adapted alongside the textual content in order to ensure consistency between the visual materials and the Spanish version of the manual. Therefore, new photographs were produced specifically for the adapted version while maintaining fidelity to the original exercises and therapeutic intent of the intervention.

A structured photographic session was conducted involving a professional photographer and two occupational therapists experienced in the implementation of the Home-GRASP program. The therapists ensured that all exercises were performed and photographed according to the original protocol, maintaining fidelity to the intervention while adapting its visual presentation to the Spanish context. Individuals appearing in the photographs provided written informed consent for the use and publication of their images in the adapted manual.

#### 2.4.4. Step 4. Pilot Testing

The preliminary Spanish version was pilot tested with stroke survivors meeting the study eligibility criteria. Pilot testing was conducted in two sequential phases. In the first phase, 10 participants were asked to review the manual and independently perform four randomly selected exercises using only the written instructions and accompanying photographs, without receiving clarification, demonstration, or assistance from the evaluator. Four exercises were assigned to each participant using a randomization procedure implemented in R statistical software. This approach was used to ensure adequate coverage of the manual while minimizing participant burden.

Participant performance was assessed by an occupational therapist using a structured observation form. For each exercise, the evaluator recorded whether the exercise was completed correctly, the occurrence of execution errors, potential causes of misunderstanding, and any comments or suggestions provided by participants regarding the clarity of the instructions or visual materials. Correct execution was defined as performance of the exercise in accordance with the written instructions, accompanying photographs, and intended movement pattern described in the manual. An execution error was defined as any observable deviation from the written instructions, visual materials, or intended movement pattern that suggested misunderstanding of the exercise. Exercise performance was video-recorded with participants’ consent to enable subsequent review when necessary. Video recordings were reviewed when clarification of observed difficulties was required and were subsequently examined by members of the research team to verify and document the issues identified during pilot testing.

Following task completion, participants completed a brief self-administered questionnaire developed specifically for this study to explore their perceptions regarding the clarity, comprehensibility, and ease of use of the translated manual. Although participants performed only selected exercises, all participants reviewed the complete manual and completed the questionnaire based on their overall perceptions of the manual as a whole. Therefore, questionnaire responses from both pilot-testing phases were pooled for analysis. The questionnaire comprised six items addressing ease of use, clarity of the instructions and visual materials, perceived need for assistance or prior professional guidance, and overall complexity of the manual. Responses were rated on a 5-point Likert scale ranging from 1 (strongly disagree) to 5 (strongly agree). Participants were also invited to provide qualitative feedback regarding wording, content, or any aspect of the manual that could be improved.

#### 2.4.5. Step 5. Development of the Final Version

Data obtained during pilot testing were independently reviewed by two members of the research team. Issues related to the interpretation of instructions, wording, or visual materials were discussed and resolved through consensus. The content and wording of the translated manual were then refined to improve linguistic clarity, cultural relevance, ease of use, and conceptual equivalence while preserving the intent and structure of the original intervention [17,18].

Following implementation of the identified modifications, the revised exercises were reassessed in an additional pilot-testing phase to confirm the adequacy of the proposed changes. The resulting version constituted the final Spanish adaptation of the Home-GRASP participant exercise manual. The final version was subsequently reviewed and approved by the developer of the original Home-GRASP program and has since been made available through the official Home-GRASP resources website.

### 2.5. Data Analysis

All analyses were performed using R statistical software version 4.5.0. (R Foundation for Statistical Computing, Vienna, Austria; http://www.r-project.org). Demographic and clinical characteristics of the participants were summarized using frequencies and percentages (n, %) for categorical variables. The distribution of continuous variables was assessed using the Shapiro–Wilk test. Normally distributed variables were reported as means and standard deviations (SD), whereas non-normally distributed variables were summarized using medians and interquartile ranges (IQR). Responses to the pilot-testing questionnaire were summarized using descriptive statistics.

## 3. Results

### 3.1. Results of the Translation and Cross-Cultural Adaptation Process

Table 1 summarizes the issues identified and the modifications implemented during the translation and cross-cultural adaptation of the Home-GRASP participant exercise manual. Comparison of the two forward translations revealed only minor differences in wording and expression. According to the expert committee, both versions conveyed the same underlying concepts and therapeutic intent. Following expert committee review of the preliminary Spanish version, several modifications were proposed to improve linguistic clarity, cultural relevance, readability, and ease of use. These included standardizing the form of address throughout the manual, replacing the original photographs with newly produced images to ensure consistency with the visual and contextual adaptations introduced in the Spanish version of the manual, and modifying the layout of the Target Board Print-Out to improve ease of use and readability. Aspects of the graphic design and exercise instructions were also revised to facilitate comprehension and correct performance. In addition, a potential barrier to task performance was identified in Exercises 5 and 6, which required participants to place the hand flat on the table while performing elbow movements. As some individuals with post-stroke upper-limb impairment may be unable to maintain this position, an alternative hand placement, with the thumb positioned beneath the table edge, was incorporated to improve stability during task performance. This modification was supported by the inclusion of an additional photograph and explanatory text box. The proposed modification was intended to facilitate task performance while preserving the therapeutic objective and movement pattern of the original exercise. The final adapted version, including this modification, was subsequently reviewed and approved by the developer of the original Home-GRASP program and is currently available through the official Home-GRASP resources website (https://neurorehab.med.ubc.ca/grasp/grasp-manuals-and-resources/; accessed on 2 June 2026).

**Figure 2:**
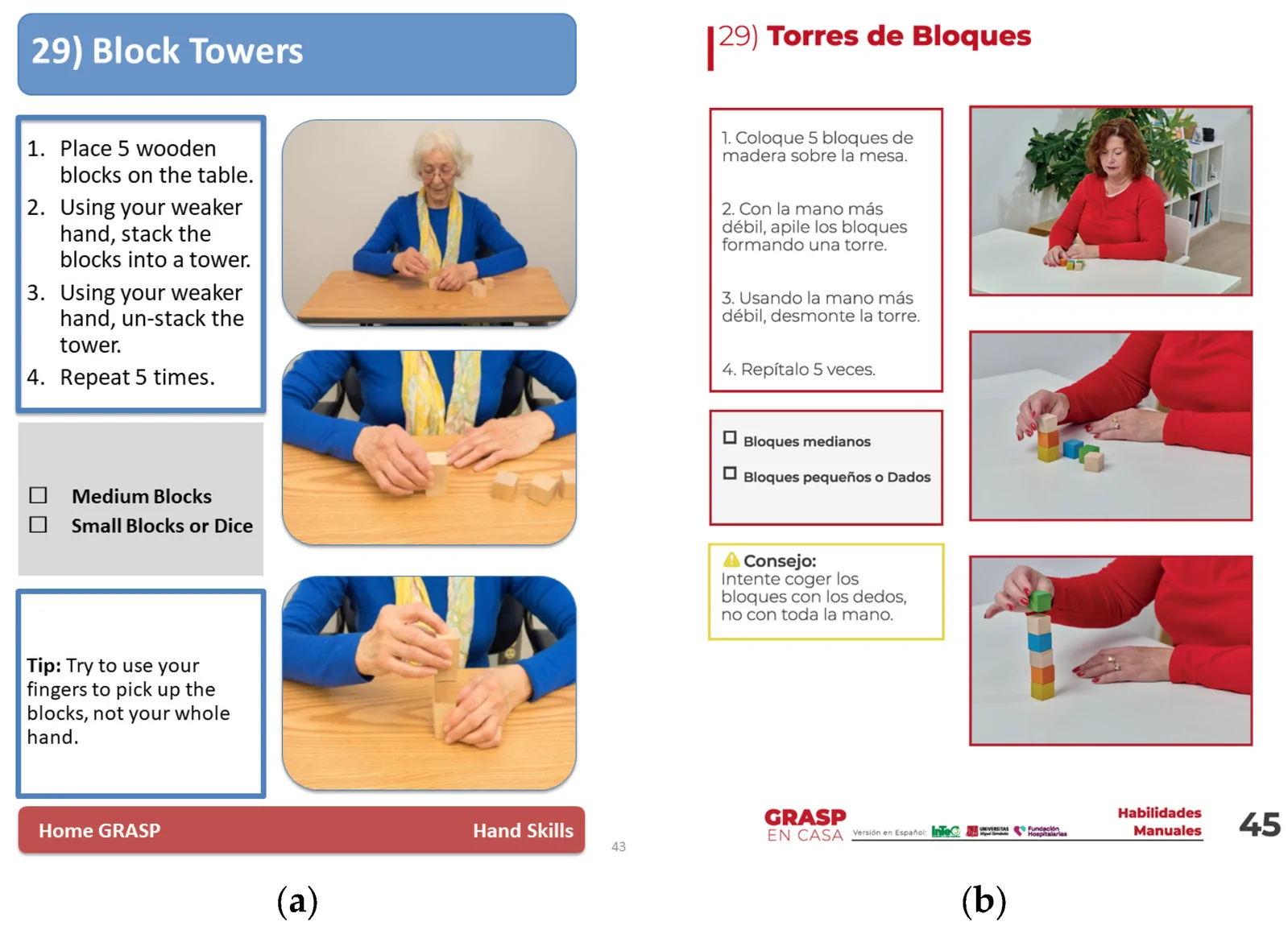
Example of Visual, Layout, and Content Adaptations Implemented During the Cross-Cultural Adaptation of the Home-GRASP Participant Exercise Manual: (**a**) Original English version of Exercise 29 (“Block Towers”); (**b**) Adapted Spanish Version (“Torres de Bloques”).

As illustrated in Figure 2, the adaptation process extended beyond linguistic translation and involved changes to the visual presentation, page layout, and photographic materials. These modifications were intended to improve readability, facilitate user identification with the material, and ensure cultural relevance while preserving fidelity to the original intervention.

### 3.2. Pilot-Testing Sample Characteristics

Table 2 presents the sociodemographic and clinical characteristics of the participants included in the pilot testing phase. The sample comprised 13 stroke survivors (7 males and 6 females) with a mean age of 55.7 years (SD = 9.0). Regarding educational attainment, 46.2% of participants reported primary education or below, whereas 53.8% reported a higher educational level. Most participants presented left-sided upper limb impairment (69.2%). The median time since stroke was 7.5 months (IQR = 4.7–9.6).

### 3.3. Pilot-Testing the Home-GRASP Participant Exercise Manual

During the initial pilot-testing phase, 10 participants independently performed four randomly assigned exercises each, resulting in a total of 40 exercise performances. All 35 exercises included in the Home-GRASP participant exercise manual were evaluated at least once, and five exercises were evaluated twice. Seven execution errors were identified, affecting four of the 35 exercises (11.4%) included in the manual. The observed difficulties were mainly related to ambiguities in movement descriptions and inconsistencies between the written instructions and accompanying photographs.

Following revision of the affected exercises, a second pilot-testing phase was conducted with three additional participants. The four revised exercises were reassessed by these participants, resulting in nine additional exercise performances. Each revised exercise was reassessed at least twice, and one exercise was reassessed three times. No further execution errors were observed after the modifications had been implemented.

In total, 49 exercise performances were evaluated across both pilot-testing phases. As shown in Table 3, most identified errors reflected misunderstandings of specific movement details rather than misinterpretations of the overall exercise objectives. These difficulties resulted in minor execution errors during independent practice and were addressed through targeted revisions to the wording and visual materials. All remaining exercises were completed without comprehension difficulties.

### 3.4. Participant-Reported Perceptions of Clarity, Comprehensibility, and Ease of Use of the Home-GRASP Participant Exercise Manual

Table 4 summarizes participants’ perceptions regarding the clarity and comprehensibility of the Home-GRASP participant exercise manual. Overall, responses indicated positive participant perceptions regarding the clarity, comprehensibility, and ease of use of the adapted manual. All participants agreed that the manual was easy to use, while 92.3% agreed that most people could complete the exercises easily and quickly. A similarly high proportion (92.3%) considered that the photographs accurately represented the exercises.

Most participants (76.9%) reported that they did not require assistance to understand and perform the exercises, and 92.3% did not consider the manual to be complex or difficult to understand. However, 53.8% indicated that they would prefer prior professional guidance before undertaking the program independently.

Qualitative feedback was consistent with the difficulties identified during exercise performance and primarily related to the wording of specific instructions. No additional concerns regarding the structure, format, visual presentation, or overall ease of use of the manual were identified.

## 4. Discussion

The present study resulted in a Spanish version of the Home-GRASP participant exercise manual that preserved the content, structure, and therapeutic intent of the original program while ensuring its linguistic and contextual suitability for stroke survivors in Spain. The adaptation process revealed a high degree of consistency between the independent forward translations, suggesting that the original content could be transferred to the target language with minimal semantic modification. Most adaptations identified by the expert committee were therefore related to contextual, visual, and ease of use aspects rather than substantial linguistic discrepancies.

One of the most relevant adaptations concerned the visual materials included in the manual. The original photographs were replaced with newly produced images to ensure consistency with the visual and contextual adaptations introduced in the Spanish version of the manual. In addition, visual supports were incorporated when difficulties in exercise execution were identified during pilot testing. These findings highlight the importance of considering visual elements as an integral component of rehabilitation interventions rather than merely supplementary materials. For stroke survivors, photographs and visual demonstrations may support the understanding and execution of therapeutic movements, particularly when written instructions involve complex movement sequences. Previous research has shown that visual observation of motor tasks can facilitate motor performance and learning after stroke [21]. Ensuring consistency between textual and visual information is therefore essential to facilitate independent exercise practice and reduce the likelihood of execution errors.

Pilot testing provided initial evidence supporting the adequacy of the adapted version. Comprehension difficulties were limited to a small number of exercises and were primarily associated with ambiguities in the description of specific movements or inconsistencies between written instructions and accompanying photographs. Importantly, these difficulties did not reflect misunderstandings of the overall objectives of the exercises but rather uncertainties regarding particular aspects of task execution. The incorporation of targeted wording clarifications and visual modifications successfully addressed these issues, resulting in a final version that was considered both clear and culturally appropriate for the target population.

Although the Home-GRASP participant exercise manual is currently available in several languages, including Chinese, Dutch, and Hungarian (https://neurorehab.med.ubc.ca/grasp/grasp-manuals-and-resources/; accessed on 2 June 2026), published descriptions of the translation and cross-cultural adaptation procedures used to develop these versions appear to be limited. Consequently, little information is available regarding the methods employed to ensure linguistic equivalence, cultural appropriateness, and ease of use across different settings. In the present study, the adaptation process extended beyond linguistic translation and included modifications to visual materials, page layout, exercise instructions, and overall ease of use of the manual. These findings support the view that the successful transfer of rehabilitation interventions across cultural contexts requires attention not only to language but also to the ways in which information is presented, interpreted, and applied by end users [22].

Of the 35 exercises included in the Home-GRASP participant exercise manual, comprehension or execution difficulties were identified in only four during pilot testing. Importantly, these difficulties were limited to specific aspects of task performance and were successfully resolved through minor modifications to the wording and visual instructions. No further execution errors were observed during subsequent reassessment of the revised exercises.

These findings are particularly relevant given that Home-GRASP is designed to support independent home-based practice by stroke survivors, with guidance provided through the participant exercise manual rather than continuous professional supervision. Consequently, the clarity of written instructions and visual materials is essential to ensure that users correctly interpret and perform the exercises as intended [22]. Difficult or ambiguous language may compromise comprehension of the intervention, reduce confidence in completing the exercises independently, and ultimately hinder adherence to the program [23]. Clear and accessible instructions are therefore fundamental not only to the ease of use of the manual but also to the faithful implementation of the intervention in everyday practice [22,23].

Previous studies have shown that stroke survivors generally perceive the GRASP program as a feasible and acceptable rehabilitation approach, with positive experiences reported across a range of rehabilitation settings [11,12]. This finding is consistent with the results of the present study, in which all participants agreed that the Home-GRASP participant exercise manual was easy to use, and 92.3% considered that most people could complete the exercises easily and quickly. Although most participants (76.9%) reported that they did not require assistance to understand or perform the exercises, a small proportion indicated that support from another person could be beneficial. This finding is not unexpected given the broad range of physical, cognitive, communicative, and functional impairments that may occur following stroke. For some individuals, assistance may be required not only to perform the exercises but also to prepare materials, organize practice sessions, interpret instructions, or ensure safe participation.

The involvement of caregivers and family members has been recognized as an important factor influencing participation in home-based rehabilitation programs following stroke [24,25,26]. Caregivers may contribute by providing practical assistance, facilitating adherence, supporting motivation, and helping integrate therapeutic activities into daily routines [24,25]. Consistent with this perspective, the Home-GRASP program was designed to encourage the active involvement of caregivers and family members whenever appropriate, promoting a collaborative approach to rehabilitation that extends beyond formal therapy sessions [13].

More than half of the participants (53.8%) indicated that they would prefer prior guidance from a healthcare professional before undertaking the program independently. Although participants generally perceived the Home-GRASP participant exercise manual as clear and easy to use, this finding highlights the importance of initial professional support when implementing home-based rehabilitation programs after stroke. A recent scoping review of home-based stroke rehabilitation interventions found that many programs do not include supervised introductory sessions or structured instruction before independent practice, despite their potential role in facilitating program engagement and correct exercise performance [27].

The preference for initial professional guidance observed in the present study may reflect the challenges that some stroke survivors face when learning new exercises, organizing practice sessions, or interpreting written and visual instructions. Early instruction may help users develop confidence in performing the exercises correctly and reduce the likelihood of errors that could lead to frustration or decreased participation. At the same time, professional support should be balanced with opportunities for independent practice. Excessively prescriptive guidance may limit problem-solving processes and adaptation of movement strategies, both of which are important components of motor learning and functional recovery after stroke [27,28]. Consistent with the original design of Home-GRASP, these findings support a model in which initial therapist guidance is used to establish correct exercise performance and confidence, followed by progressively autonomous home-based practice [13].

Several limitations should be considered when interpreting the findings of this study. Although the pilot-testing sample was consistent with recommendations for the cross-cultural adaptation of health-related materials [29], most exercises were initially performed by only one participant, while a small number were performed twice. Consequently, some less frequent comprehension or execution difficulties may have remained undetected. Furthermore, the absence of observed difficulties in a specific exercise should not be interpreted as definitive evidence that the exercise will be consistently understood and performed correctly by all users. Therefore, the findings regarding clarity, comprehensibility, and ease of use should be interpreted as preliminary evidence derived from pilot testing rather than as conclusive evidence applicable to all potential users of the manual. In addition, the questionnaire used to collect participant feedback was developed specifically for this study and did not undergo formal psychometric validation. Consequently, the questionnaire findings should be interpreted as participant-reported perceptions regarding clarity, comprehensibility, and ease of use rather than as a formal assessment of ease of use. Another limitation was that participants were recruited from a single rehabilitation center, which may have limited the range of perspectives represented during the adaptation process. Pilot testing was also conducted in a supervised clinical setting rather than in participants’ home environments, where factors such as distractions, available space, and access to materials may influence exercise performance and program use. Participants were also selected if they had sufficient upper-limb motor function and cognitive abilities to engage with the manual and perform the exercises independently. Therefore, the findings may not be generalizable to stroke survivors with more severe motor, cognitive, language, or perceptual impairments. In addition, clinical characteristics such as stroke type, aphasia status, severity of upper-limb impairment, and perceptual deficits were not systematically collected. These factors may influence the interpretation and independent execution of written or visually demonstrated exercises and should be considered in future research. Moreover, the adapted version was developed and pilot tested exclusively in Spain. Therefore, the findings should be interpreted within the context of the Spanish population included in this study. Although the manual is written in Spanish and may be understandable in other Spanish-speaking settings, its cultural suitability and ease of use in other countries and healthcare contexts remain to be established. Further research is required to evaluate its applicability in other Spanish-speaking populations. Finally, this study focused on the translation and cross-cultural adaptation of the manual and did not evaluate the feasibility, effectiveness, adherence, or long-term implementation of the Home-GRASP program in Spanish-speaking populations.

Despite these limitations, the study has several strengths. The translation and cross-cultural adaptation process followed a systematic methodology that included independent forward translations, expert committee review, adaptation of visual materials, and pilot testing with representatives of the target population. In addition to assessing participants’ perceptions of clarity and comprehensibility, the study incorporated direct observation of exercise performance. This approach enabled the identification of execution difficulties and ambiguities that may not have been detected through self-report measures alone. Furthermore, the inclusion of adapted visual materials and the evaluation of exercise execution under conditions of independent use contributed to the development of a version that preserves the intent of the original intervention while enhancing its clarity, ease of use, and suitability for stroke survivors in Spain.

## 5. Conclusions

This study provides the first Spanish translation and cross-culturally adapted version of the Home-GRASP participant exercise manual. The adaptation process preserved the content and therapeutic intent of the original program while ensuring its linguistic and contextual appropriateness for use in Spain. Pilot testing provided preliminary evidence supporting the clarity, comprehensibility, and ease of use of the adapted manual, with only minor modifications required following participant feedback and observation of exercise performance. The resulting Spanish version constitutes a linguistically and contextually adapted resource that may support the future implementation of Home-GRASP in rehabilitation settings in Spain and facilitate home-based upper-limb practice after stroke. Future research should evaluate the feasibility, acceptability, and effectiveness of the Spanish version in real-world clinical and community contexts, as well as its cultural appropriateness and ease of use in other Spanish-speaking countries.

## Figures and Tables

**Table 1:** Issues identified during the translation and cross-cultural adaptation of the Home-GRASP participant exercise manual and modifications implemented by the expert committee.

Issue Identified	Modification Implemented
The original manual uses direct second-person forms (“you”) and inclusive first-person plural expressions (“we”), which do not have a direct equivalent in Spanish and may sound unnatural in certain contexts.	The Spanish version was standardized using the formal form of address (“usted”) throughout the manual to ensure linguistic consistency, sociocultural appropriateness, and natural readability.
When the manual is printed double-sided, the Target Board Print-Out may not be usable because both pages of the board can appear on opposite sides of the same sheet.	A blank page was inserted after the cover page to ensure that the Target Board Print-Out can be printed as a separate removable resource when the manual is produced in double-sided format.
The photographs included in the original version required review to ensure consistency with the linguistic, contextual, and visual adaptations introduced in the Spanish version.	All photographs were replaced with newly produced images to ensure consistency with the linguistic, contextual, and visual adaptations introduced in the Spanish version while maintaining fidelity to the original exercises.
Some design elements and page layouts were considered difficult to follow, potentially affecting readability and ease of use.	The visual design and layout of the manual were revised to improve readability, visual organization, and user experience.
In Exercises 5 and 6, the hand position shown in the original photographs may be difficult to reproduce for individuals with post-stroke upper-limb impairment, potentially affecting correct performance of the exercises.	New photographs and additional instructions were incorporated to illustrate an alternative hand position that facilitates grip stability and correct execution of the exercises.

**Table 2:** Sociodemographic and clinical characteristics of participants included in the pilot testing phase (n = 13).

Characteristics	n (%)
Sex	
Male	7 (53.8)
Female	6 (46.2)
Age (years), mean (SD)	55.7 (9.0)
Educational level	
Primary education or below	6 (46.2)
Above primary education	7 (53.8)
Most affected upper limb	
Right	4 (30.8)
Left	9 (69.2)
Time since stroke (months), median (IQR)	7.5 (4.7–9.6)

Abbreviations: SD, standard deviation; IQR, interquartile range.

**Table 3:** Exercise-specific difficulties identified during pilot testing and corresponding modifications implemented.

Exercise	Observed Difficulty	Modification Implemented
5 (Push-ups)	Two participants misinterpreted the movement sequence. One participant used the arms to raise the body without bending the knees, whereas another did not bend the knees when returning to the starting position. These difficulties suggested that the description of the movement performed against the table was insufficiently explicit.	To improve clarity, the phrase “as if performing a push-up” was added to the exercise instructions. Following this modification, no further execution difficulties were observed.
6 (One-arm push-ups)	One participant did not return to the starting position after completing the movement. Another participant completed the exercise correctly but reported difficulty understanding the instructions.	Consistent with Exercise 5, the phrase “as if performing a push-up” was incorporated into the instructions to clarify the intended movement pattern. No further execution difficulties were observed after revision.
15 (The twist)	One participant manipulated the putty without stretching it, while another used different fingers across repetitions instead of using the first three fingers simultaneously.	The exercise description was revised to explicitly state that the putty should be stretched and that the first three fingers should be used “at the same time.” Following these modifications, participants performed the exercise correctly.
33 (Flip over)	One participant turned the poker chips without performing the required forearm supination movement, indicating that the intended movement was not sufficiently explicit in the instructions.	The phrase “turn the arm face-up” was added to the instructions to clarify the required movement. No further execution difficulties were observed following implementation of the revision.

**Table 4:** Participant perceptions of the clarity, comprehensibility, and ease of use of the Home-GRASP participant exercise manual (n = 13).

	Strongly Disagree	Disagree	Neither	Agree	Strongly Agree
Ease of use of the manual	0	0	0	5 (38.5)	8 (61.5)
Need for assistance to understand and perform the exercises	7 (53.8)	3 (23.1)	1 (7.7)	2 (15.4)	0
Perceived ease and speed of completing the exercises	0	1 (7.7)	0	4 (30.8)	8 (61.5)
Need for prior professional guidance	1 (7.7)	3 (23.1)	2 (15.4)	6 (46.1)	1 (7.7)
Clarity of the photographs	0	0	1 (7.7)	2 (15.4)	10 (76.9)
Perceived complexity of the manual	6 (46.1)	6 (46.1)	1 (7.7)	0	0

Data are presented as n (%). Neither = neither agree nor disagree.

## Data Availability

The dataset used and analyzed during the current study is available from the corresponding author upon reasonable request. Data are not publicly available due to ethical and legal restrictions imposed by the Ethics Committee of the General University Hospital of Alicante. In the informed consent process, participants were assured that their personal information and study data would remain confidential.

## Abbreviations

The following abbreviations are used in this manuscript:

GRASP	Graded Repetitive Arm Supplementary Program
Home-GRASP	Home Graded Repetitive Arm Supplementary Program
IQR	Interquartile range
SD	Standard deviation
